# Malnutrition among Children under Age Five in Panama: Results of the ENSPA 2019

**DOI:** 10.5334/aogh.4409

**Published:** 2024-08-08

**Authors:** Alicia Sosa Pedreschi, Flavia Fontes, Reina Roa, Hedley Quintana, Roger Montenegro Mendoza

**Affiliations:** 1 Department of Research and Health Technology Assessment, Gorgas Memorial Institute for Health Studies, Panama; 2 Dietetic and Nutrition Department, Faculty of Medicine, University of Panama, Panama; 3 Planning Directorate, Ministry of Health, Panama; 4 Preventive and Social Medicine Department, Faculty of Medicine, University of Panama, Panama

**Keywords:** Undernutrition, overnutrition, double burden of malnutrition, Panama

## Abstract

*Background:* Malnutrition has important short- and long-term consequences in children under age five. Malnutrition encompasses undernutrition, overnutrition, and the coexistence of both of them, known as the double burden of malnutrition (DBM).

*Objective:* The aim of this study was to estimate the prevalence of undernutrition, overnutrition, and the DBM among these children at the national level and by living area in Panama.

*Methods:* Data from the National Health Survey of Panama (ENSPA, Spanish acronym), a population-based, cross-sectional study carried out in 2019 were used. Stunting, wasting, overweight, and obesity were defined according to the cut-off points of the World Health Organization Growth Standards. Undernutrition was defined as being stunted only, wasted only or both; overnutrition was defined as being overweight only or obese only; and the DBM was defined as the co-occurence of stunting and overweight/obesity in the same child. Prevalence and general characteristics at the national level and by living area were weighted.

*Findings:* The prevalence of undernutrition was 15.3% (95% confidence interval (CI) 13.4–17.3) at the national level and 36.6% (CI: 30.1–43.5) in indigenous areas. The prevalence of overnutrition was 10.2% (8.2–12.6) at the national level and 11.9% (CI: 8.5–16.3), 8.4% (CI: 6.5–10.7) and 8.7% (CI: 5.2–14.3) in urban, rural and indigenous areas, respectively. The DBM prevalence was 1.4% (CI: 1.0–2.1) at the national level and 2.7% (CI: 1.4–5.1) in indigenous areas.

*Conclusions:* Undernutrition is still the most prevalent malnutrition condition in our country. Panama has the highest prevalence of overnutrition in Central America. The highest prevalence of undernutrition and DBM was found among children living in indigenous areas.

## Introduction

Malnutrition is an important global public health problem. It is responsible for 45% of deaths among children under age five [1]. Malnutrition encompasses both undernutrition and overnutrition, and over the last decades, another important nutritional problem has emerged: the double burden of malnutrition (DBM). The DBM refers to the coexistence of undernutrition and excess body weight and can be studied at the population, household, or individual level.

Wasting and stunting are forms of undernutrition, with a global prevalence among children under age five of 6.8% (45 million) and 22.3% (148.1 million), respectively [2]. Wasting is the result of recent energy restriction or episodes of infection and indicates the current nutritional status of the child. Wasting increases the risk of death among children aged under age five, and recurrent periods of wasting increase the risk of stunting [1]. Stunting indicates chronic undernutrition. In the short term, stunted children have an increased morbidity and mortality risk, delayed and permanent cognitive and motor development impairments, and a reduction in schooling [3]. Furthermore, long-term consequences of stunting include increased susceptibility to central obesity, increased risk of cardiovascular and metabolic diseases in adulthood, and lower economic productivity [3].

On the other hand, overnutrition refers to the presence of overweight or obesity, with a global joint prevalence of 5.6% (37 million) [2]. Overweight and obesity are associated with excessive energy intake. Obesity has immediate repercussions on children’s health, such as respiratory and metabolic abnormalities. Moreover, obese children are five times more likely than non-obese children to become obese adults and therefore are at higher risk of developing cardiovascular and metabolic diseases such as hypertension, dyslipidaemia, type 2 diabetes mellitus, and metabolic syndrome, according to published reviews and studies [4].

In Panama, previous analysis from nationally representative data, has shown that there is already a high prevalence of overweight, obesity, central obesity, and cardiovascular diseases among adults, which has important repercussions on public health and the economy [5, 6].

The DBM at the individual level (among children younger than age five and based solely on anthropometric assessments of nutritional status) consists of stunted children who are also overweight or obese. This appears to be due to a combination of metabolic adaptations to prolonged periods of undernutrition that are followed by a shift in nutritional practices, creating an overload on the metabolic system and thus affecting homeostasis [7].

Although the global prevalence of undernutrition among children younger than 5 years has decreased, it remains a concern [2]. In 2008, the Panamanian national wasting and stunting prevalences were 1.2% and 19.1%, respectively [8]. On the other hand, the national prevalences of overweight and obesity were 8.5% and 2.8%, respectively [8].

The monitoring of the DBM has become more relevant due to the global nutritional transition, which is occurring as a consequence of economic growth. The nutritional transition is characterized by higher intakes of energy-dense food, saturated fats, and sugars and decreased intakes of micronutrients and fibre-rich foods [9]. As Panama has experienced exponential economic growth in recent decades, a nutritional transition is expected to occur. However, to the best of our knowledge, the DBM has not been studied in Panama thus far.

Panama’s efforts in malnutrition eradication are supported by its commitment to the Sustainable Development Goals for 2030 and the Community of Latin American and Caribbean States (CELAC) 2025 targets [10, 11], as well as the development of the Panama Food and Nutritional Security National Plan 2017–2021 as a framework to ensure meeting these commitments [12]. However, continuous monitoring of nutritional status is needed to identify vulnerable groups, update policies, and develop strategies in a timely fashion based on the current national status. Moreover, socioeconomic and health intervention coverage inequalities have been previously described in Panama as affecting people from indigenous areas and thus requiring special attention to this population [13, 14].

Therefore, the aim of the present study was to estimate the prevalence of undernutrition, overnutrition, and the DBM among children under age five at the national level and according to living area in Panama.

## Materials and Methods

### Settings

The National Health Survey of Panama (ENSPA, Spanish acronym) 2019 was a nationwide, population-based, cross-sectional study designed to assess general health status, the prevalence of several diseases, and the possible associated risk factors in the Panamanian population. A complex sample design (randomized, tri-phased, and stratified by conglomerates) was used to select households and participants to achieve representativeness of the results at the country level, including urban, rural, and indigenous areas. For children under 5 years old, additional primary sampling units that contained the target population were intentionally selected within the domains of the ENSPA, given the low Panamanian fertility rate. Data collection involved an extensive questionnaire, anthropometric and blood pressure measurements, and blood sampling. Between June and December 2019, from each household one individual ≥15 years old and, if available, one individual <15 years old were randomly selected to be evaluated. In total, 17,997 individuals ≥15 years old and 10,486 individuals <15 years old were enrolled in the study. The ENSPA 2019 has been previously described [5, 6], and a detailed description is found on their website in the Spanish language [15].

For the present study, data from children under age five were retrieved from the <15-year-old questionnaire, and household information were retrieved from their household questionnaire of the ENSPA 2019 study.

### Participants

In this study, we excluded from the analyses children with missing or invalid information on anthropometric data, those who had oedema, and preterm children.

### Sociodemographic characteristics

Age was measured in months, and sex assigned at birth was self-reported as male or female. Living area was divided into urban, rural, and indigenous. Overcrowding was defined as more than three people per habitable room within a household. Household monthly income quartiles were calculated based on the household’s monthly incomes from this study sample: first quartile (≤124 USD), second quartile (125–399 USD), third quartile (400–699 USD), and fourth quartile (≥700 USD). The household dietary diversity (HDD) score was defined as the number of food groups—cereals, roots and tubers, legumes, vegetables, fruits, egg, meat, seafood and fish, dairy, fats, natural condiments, and sugars—eaten over the previous 24 hours by any member of the household. For each group eaten by any member of the household, a point was awarded. HDD was divided into three levels according to the score as follows: low diversity (0–3 points), medium diversity (4–6 points), and high diversity (7–12 points).

### Water, sanitation, and hygiene characteristics

Water, sanitation, and hygiene (WASH) characteristics included drinking water–source status, sanitation system, and adequate hygiene. Drinking water source was defined as the principal source from which drinking water was obtained and was categorized into improved or unimproved according to its degree of safety, based on the World Health Organization (WHO) Joint Monitoring Programme (JMP) for Water Supply, Sanitation and Hygiene 2017 [16]. Improved drinking water source included the particular aqueduct, community public aqueduct, protected dug well, and bottled water. Unimproved drinking water sources included unprotected dug wells, artisan wells, rivers, streams, lakes, ponds, community water taps, rainwater, tanker trucks, spring water, or other sources. For drinking water sources, a modification of WHO classifications was applied, considering community water tap, rainwater, and tanker truck as unimproved sources due to a lack of information regarding adequate storage.

The “sanitation system” referred to the method used for managing or disposing of waste from households and was categorized as improved or unimproved depending on the level of safety [16]. An improved sanitation system included sewage and septic tanks. An unimproved sanitation system included a latrine and the absence of a sanitation system or any other system.

For adequate hygiene, hand washing before eating and after toilet use was evaluated. If the person responded “always” to both questions, then hygiene was considered adequate. If the person responded with a lower frequency (never, rarely, sometimes, or almost always) to any of the corresponding queries, hygiene was considered inadequate.

### Access to health care

Access to health care was evaluated based on responses about growth checkups compliance. Adequacy of growth checkups was established by age as follows:

- Children under one month: one checkup in the first month of life
- Between 1 and 2 months: two checkups in total
- Between 3 and 5 months: three checkups in total
- Between 6 and 7 months: four checkups in total
- Between 8 and 11 months: five checkups in total
- From 12 months to 59 months: one checkup in the last year [17].

### Comorbidities

Information about the occurrence of diarrhoea and acute respiratory infections in the last 2 weeks was collected from the questionnaire.

### Anthropometric measurements

Anthropometric measurements were performed in the household of the participants at the time of the survey. The anthropometric evaluation included weight, using a portable digital scale SECA Model 874dr, and height, using a stadiometer SECA Model 213I equipped with a level (Hamburg, Germany), or length, using an infantometer SECA Model 417 with a solid measurement board that was foldable and portable with a fixed stop for the head and a mobile stop for the feet. Weight was measured with a precision of 0.5 kg, while height/length was measured with a precision of 0.1 mm.

For children under 2 years old, weight was determined using the two-in-one function of the scale that allowed us to weigh children held in their parent’s arms. Length was measured in the lying down position, with the infantometer for children under 2 years old, and for children aged 2 years and older, height was assessed in a standing position with the stadiometer.

For all anthropometric measurements, two repeated measurements were performed by two trained health personnel, with an absolute maximal difference accepted between measurements of 0.5 kg for the weight and 0.5 cm for the height/length. When the difference between the two measurements was greater than the maximal error accepted, a third measurement was performed, and the average of the two closest measurements was recorded and used for the analysis.

### Malnutrition indicators

Malnutrition was defined as the presence of wasting, stunting, overweight, or obesity. “Wasting” was defined as a weight-for-height/length Z score below −2 standard deviations (SDs) from the median value of the WHO Growth Standards; “stunting” was defined as a height/length-for-age Z score below −2 SDs from the median value; and “overweight” and “obesity” were defined as a weight-for-height/length Z score above +2 and +3 SDs, respectively [18].

Calculation of Z scores of anthropometric nutritional status indicators was performed using the macro for SPSS (IBM SPSS Statistics, version 25). Height/length-for-age Z scores below −6 and above +6 and weight-for-height/length Z scores below −5 and above +5 were considered extreme values and were excluded from the analyses.

### Undernutrition

“Undernutrition” was defined as the presence of stunting only, wasting only, or the coexistence of both at the individual level.

### Overnutrition

“Overnutrition” was defined as being overweight only or obese only at the individual level.

### Double burden of malnutrition

The DBM was evaluated at the individual level using anthropometric indicators, indicating the presence of both stunting and overweight or obesity in the same child.

### Statistical analysis

Categorical variables are presented as percentages and their 95% confidence intervals (CIs). Prevalence and general characteristics were weighted and used a complex sampling design.

All calculations were performed using SPSS software.

### Statement of ethics

Ethical approval to develop the ENSPA 2019 study was obtained from the Ethics Review Committee of the Gorgas Memorial Institute for Health Studies (749/CBI/ICGES/9 August 2017). All participants were informed about the objectives of the ENSPA 2019, and the caregivers of the children were given written informed consent forms. The present study is a secondary analysis of the open-access ENSPA 2019 database.

## Results

**Figure 1:**
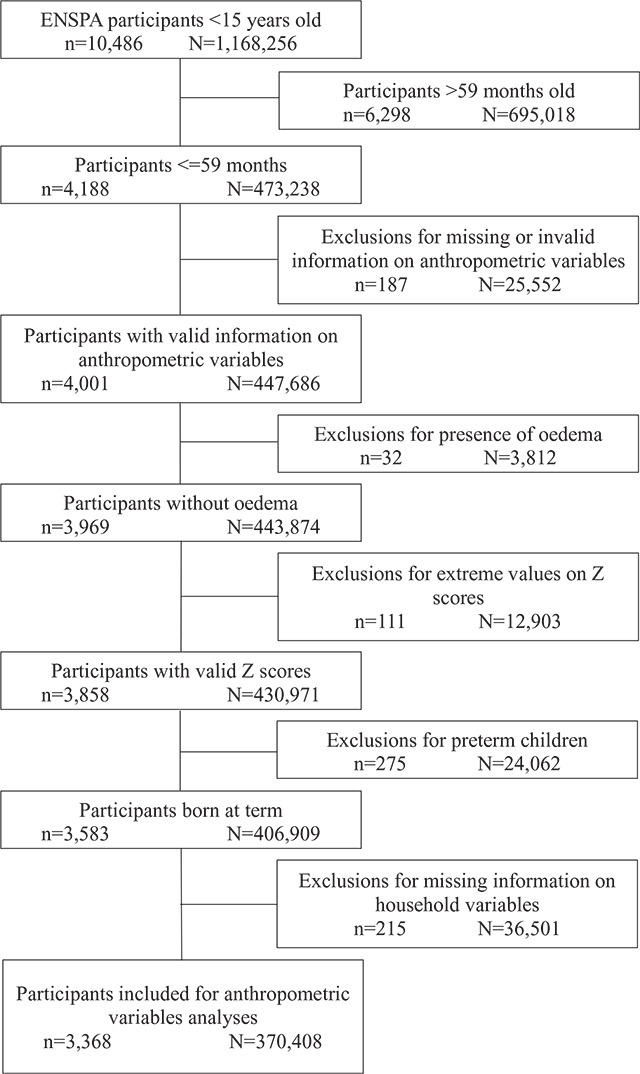
Flowchart summarizing the inclusion and exclusion criteria for participation.

Figure 1 presents a flowchart of the participants, following the inclusion and exclusion criteria. The weighted sample for the present study represented 370,408 children under age five nationwide. Supplementary Table 1 presents the baseline characteristics (age, sex, and living area) of the excluded children.

The weighted baseline characteristics of children under age five at the national level and by living area are presented in Table 1. In indigenous areas, two out of five children lived in overcrowded households (41.2%; 95% CI: 34.4–48.3), but the figure for children living in urban (8.4%; 95% CI: 6.3–11.0) and rural (10.3%; 95% CI: 8.2–12.8) areas was approximately 1 out of 10. Three out of four children living in indigenous areas (77.6%; 95% CI: 69.5–84.1) had a household monthly income in the first quartile (≤124 USD). At the national level, three out of four children (75.2%; 95% CI: 72.5–77.8) lived in a household with a high HDD. However, two out of three children living in indigenous areas had either a low (24.0%; 95% CI: 18.5–30.6) or a medium (35.6%; 95% CI: 28.5–43.3) HDD. Nationally, approximately one-third of the children lived in a household with an unimproved sanitation system (34.3%; 95% CI: 31.5–37.2), and nearly half of them (53.5%; 95% CI: 49.8–57.2) had inadequate hygiene practices. The vast majority (93.6%; 95% CI: 88.3–96.6) of the children from indigenous areas had unimproved sanitation systems.

**Table 1:** Selected baseline characteristics of the children under age five at the national level and by living area, Panama, 2019.

SELECTED BASELINE CHARACTERISTICS	LIVING AREA							
	NATIONAL		URBAN AREA		RURAL AREA		INDIGENOUS AREA	
	N	WEIGHTED PREVALENCE % (95% CI)	N	WEIGHTED PREVALENCE % (95% CI)	N	WEIGHTED PREVALENCE % (95% CI)	N	WEIGHTED PREVALENCE % (95% CI)
Total	370,408	100.0	184,798	49.9 (46.7–53.0)	124,196	33.5 (30.8–36.3)	61,414	16.6 (14.4–19.0)
**Sociodemographic characteristics**								
**Age (months)**								
0–11	50,317	13.6 (11.7–15.7)	24,303	13.2 (10.3–16.7)	17,228	13.9 (11.3–16.9)	8,786	14.3 (10.2–19.7)
12–23	75,137	20.3 (17.9–23.0)	36,031	19.5 (15.8–23.8)	25,582	20.6 (17.5–24.1)	13,524	22.0 (15.8–29.9)
24–35	85,444	23.1 (20.5–25.9)	38,879	21.0 (17.1–25.6)	28,759	23.2 (19.6–27.1)	17,805	29.0 (22.8–36.1)
36–47	80,472	21.7 (19.4–24.2)	46,949	25.4 (21.6–29.7)	24,490	19.7 (16.7–23.1)	9,033	14.7 (10.9–19.5)
48–59	79,038	21.3 (18.7–24.3)	38,635	20.9 (17.3–25.1)	28,136	22.7 (18.1–27.9)	12,266	20.0 (13.8–27.9)
**Female sex**	169,158	45.7 (42.5–48.8)	76,020	41.1 (36.5–45.9)	62,110	50.0 (45.4–54.6)	31,027	50.5 (42.9–58.1)
**Overcrowding**	53,454	14.4 (12.7–16.3)	15,436	8.4 (6.3–11.0)	12,737	10.3 (8.2–12.8)	25,281	41.2 (34.4–48.3)
**Household monthly income quartile (USD; $)**								
First quartile (≤124)	101,824	29.2 (26.6–32.0)	18,679	10.8 (8.4–16.6)	37,233	32.0 (28.1–36.3)	45,911	77.6 (69.5–84.1)
Second quartile (125–399)	94,054	27.0 (24.2–30.0)	45,205	26.1 (21.8–30.9)	37,469	32.2 (28.2–36.5)	11,380	19.2 (13.0–27.6)
Third quartile (400–699)	83,150	23.8 (21.1–26.8)	54,117	31.2 (27.1–35.6)	27,478	23.6 (18.8–29.3)	1,555	2.6 (1.5–4.4)
Fourth quartile (≥700)	69,765	20.0 (17.3–23.0)	55,405	32.0 (27.3–37.0)	14,064	12.1 (9.5–15.3)	296	0.5 (0.2–1.1)
**Household dietary diversity**								
Low (0–3)	28,576	7.7 (6.1–9.7)	7,653	4.1 (2.2–7.8)	6,182	5.0 (3.6–6.8)	14,740	24.0 (18.5–30.6)
Medium (4–6)	63,164	17.1 (14.9–19.4)	21,696	11.7 (9.1–15.0)	19,627	15.8 (13.0–19.0)	21,840	35.6 (28.5–43.3)
High (7–12)	278,668	75.2 (72.5–77.8)	155,449	84.1 (80.0–87.5)	98,386	79.2 (75.7–82.3)	24,833	40.4 (33.0–48.3)
**WASH characteristics**								
**Unimproved drinking water source status**	47,651	12.9 (11.0–15.0)	8,206	4.4 (3.0–6.5)	16,649	13.4 (10.9–16.4)	22,797	37.1 (29.8–45.1)
**Unimproved sanitation system**	127,008	34.3 (31.5–37.2)	22,327	12.1 (9.5–15.2)	47,197	38.0 (33.8–42.4)	57,484	93.6 (88.3–96.6)
**Inadequate hygiene**	153,547	53.5 (49.8–57.2)	75,800	51.1 (45.9–57.1)	46,406	49.0 (43.6–54.6)	31,340	69.1 (60.9–76.3)
**Access to health care**								
**Inadequate growth checkups**	84,670	22.9 (20.4–25.6)	41,737	22.6 (18.7–27.0)	27,143	21.9 (18.7–25.4)	15,790	25.7 (19.7–32.8)
**Comorbidities**								
**Diarrhoea**	37,347	10.3 (8.7–12.1)	14,129	7.8 (5.6–10.7)	11,123	9.2 (7.2–11.6)	12,095	20.1 (15.5–25.6)
**Acute respiratory infections**	107,648	29.1 (26.1–32.2)	55,642	30.1 (25.5–35.1)	35,974	29.0 (24.4–34.0)	16,032	26.1 (21.0–31.9)

Source: National Health Survey of Panama (ENSPA) 2019; CI: confidence interval; Twenty-one thousand six hundred fourteen (21,614) participants had missing values or did not know their household monthly income. Ninety-six (96) participants had missing values for sanitation system variable. Eighty-three thousand three hundred forty-seven (83,347) participants had missing values for adequate hygiene variable. Seven thousand nine hundred six (7,906) participants had missing values for the diarrhoea in the last two weeks’ variable.

Table 2 presents the weighted prevalence of undernutrition among children under age five by baseline characteristics at the national level and by living area. Children living in indigenous areas had between a 3.5- and a 3.1- times-higher prevalence of undernutrition (36.6%; 95% CI: 30.1–43.5) compared to those living in urban (10.5%; 95% CI: 8.2–13.4) and rural (11.8%; 95% CI: 9.5–14.7) areas, respectively. At the national level, undernutrition prevalence was the highest among children aged between 12 and 23 months (18.6%; 95% CI: 14.5–23.6) and among those aged between 24 and 35 months (17.5%; 95% CI: 13.6–22.3). The prevalence of undernutrition in indigenous areas was significantly higher among children aged 24 months or older than among their younger peers. Overall, boys (16.5%; 95% CI: 13.9–19.5) had a higher prevalence of undernutrition than girls (13.8%; 95% CI: 11.5–16.4). The prevalence of undernutrition was higher among children living in households with overcrowding (29.8%; 95% CI: 24.7–35.4), with lower monthly incomes (first quartile: 25.2%; 95% CI: 21.2–29.6), with a lower HDD (low HDD: 25.1%; 95% CI: 17.7–34.2), and with unimproved drinking water sources (25.3%; 95% CI: 19.8–31.8) and sanitation systems (25.3%; 95% CI: 21.8–29.2) than among those living with better household conditions. Moreover, the prevalence of undernutrition among children with episodes of diarrhoea in the 2 weeks prior to the survey was almost twice (27.3%; 95% CI: 20.0–36.0) that among children without diarrhoea (13.8%; 95% CI: 12.0–15.8). The prevalence of stunting only, wasting only, and the coexistence of stunting and wasting are presented in Supplementary Table 2.

**Table 2:** Weighted prevalence of undernutrition among children under age five by baseline characteristics at the national level and by living area, Panama, 2019.

SELECTED BASELINE CHARACTERISTICS	LIVING AREA							
	NATIONAL		URBAN AREA		RURAL AREA		INDIGENOUS AREA	
	N	% (95% CI)	N	% (95% CI)	N	% (95% CI)	N	% (95% CI)
Total	370,408	15.3 (13.4–17.3)	184,798	10.5 (8.2–13.4)	124,196	11.8 (9.5–14.7)	61,414	36.6 (30.1–43.5)
**Sociodemographic characteristics**								
**Age (months)**								
0–11	50,317	9.6 (6.4–14.1)	24,303	8.6 (4.4–16.3)	17,228	9.9 (5.1–18.4)	8,786	11.5 (5.6–22.3)
12–23	75,137	18.6 (14.5–23.6)	36,031	17.2 (11.0–25.9)	25,582	10.5 (6.8–15.9)	13,524	26.8 (16.5–40.3)
24–35	85,444	17.5 (13.6–22.3)	38,879	9.3 (5.5–15.4)	28,759	11.6 (7.6–17.3)	17,805	46.8 (34.1–60.0)
36–47	80,472	14.2 (10.6–18.8)	46,949	9.7 (5.4–16.7)	24,490	11.6 (7.6–17.3)	9,033	44.9 (31.0–59.6)
48–59	79,038	14.3 (10.6–18.9)	38,635	7.5 (3.9–14.0)	28,136	10.5 (5.3–19.7)	12,266	44.2 (27.1–62.9)
**Sex**								
Male	201,250	16.5 (13.9–19.5)	108,778	11.3 (8.0–15.7)	62,085	13.1 (10.1–16.8)	30,386	42.3 (33.8–51.4)
Female	169,158	13.8 (11.5–16.4)	76,020	9.4 (6.7–13.0)	62,110	10.5 (7.2–15.2)	31,027	30.9 (22.7–40.5)
**Overcrowding**								
Yes	53,454	29.8 (24.7–35.4)	15,436	17.4 (9.3–30.1)	12,737	14.0 (8.7–21.9)	25,281	45.3 (37.0–53.8)
No	316,953	12.8 (10.9–15.0)	169,362	9.9 (7.5–12.9)	111,458	11.6 (9.1–14.7)	36,133	30.5 (22.0–40.5)
**Household monthly income quartile (USD; $)**								
First quartile (≤124)	101,824	25.2 (21.2–29.6)	18,679	8.6 (4.2–16.8)	37,233	17.0 (12.0–23.6)	45,911	38.5 (31.0–46.6)
Second quartile (125–399)	94,054	14.2 (11.2–18.0)	45,205	11.5 (7.4–17.5)	37,469	13.3 (9.2–18.7)	11,380	28.1 (16.0–44.5)
Third quartile (400–699)	83,150	11.5 (7.9–16.3)	54,117	13.4 (8.5–20.5)	27,478	6.4 (3.5–11.4)	1,555	32.6 (15.7–55.7)
Fourth quartile (≥700)	69,765	6.9 (4.3–10.8)	55,405	6.9 (4.0–11.9)	14,064	5.4 (2.2–12.9)	296	60.0 (24.2–87.6)
**Household dietary diversity**								
Low (0–3)	28,576	25.1 (17.7–34.2)	7,653	1.1 (0.1–7.9)	6,182	19.4 (10.9–32.3)	14,740	39.8 (27.8–53.2)
Medium (4–6)	63,164	23.7 (18.8–29.5)	21,696	16.2 (9.3–26.6)	19,627	17.6 (10.0–29.0)	21,840	36.7 (26.5–48.2)
High (7–12)	278,668	12.3 (10.4–14.6)	155,449	10.2 (7.7–13.3)	98,386	10.2 (8.0–13.0)	24,833	34.5 (24.5–46.2)
**WASH characteristics**								
**Drinking water source status**								
Unimproved	47,651	25.3 (19.8–31.8)	8,206	15.9 (6.7–33.4)	16,649	12.2 (6.7–21.4)	22,797	38.3 (27.5–50.4)
Improved	322,756	13.8 (11.9–15.9)	176,592	10.2 (7.9–13.2)	107,548	11.8 (9.3–14.8)	38,617	35.5 (27.8–44.1)
**Sanitation system**								
Unimproved	127,008	25.3 (21.8–29.2)	22,327	14.6 (7.8–25.7)	47,197	17.1 (12.5–23.0)	57,484	36.2 (29.8–43.1)
Improved	243,303	10.0 (8.1–12.3)	162,375	9.9 (7.6–12.9)	76,999	8.6 (6.4–11.4)	3,929	41.7 (14.3–75.4)
**Adequate Hygiene**								
Yes	133,514	12.3 (9.7–15.6)	71,314	11.4 (7.8–16.4)	48,206	7.7 (4.9–11.7)	13,994	33.2 (22.4–46.1)
No	153,547	16.7 (13.8–20.0)	75,800	9.5 (6.1–14.6)	46,406	13.7 (9.6–19.2)	31,340	38.2 (28.8–48.6)
**Access to health care**								
**Adequate growth checkups**								
Yes	285,738	15.3 (13.2–17.6)	143,061	10.9 (8.2–14.3)	97,052	11.4 (8.8–14.7)	45,624	37.0 (29.5–45.2)
No	84,670	15.3 (11.9–19.5)	41,737	9.1 (5.4–15.0)	27,143	13.2 (8.7–19.6)	15,790	35.2 (23.7–48.7)
**Comorbidities**								
**Diarrhoea**								
Yes	37,347	27.3 (20.0–36.0)	14,129	25.8 (13.0–44.9)	11,123	6.2 (3.0–12.4)	12,095	48.4 (36.1–61.0)
No	325,154	13.8 (12.0–15.8)	166,754	9.3 (7.2–12.0)	110,387	12.5 (9.9–15.7)	48,013	32.3 (25.4–40.2)
**Acute respiratory infections**								
Yes	107,648	16.3 (13.0–20.1)	55,642	9.5 (5.8–15.2)	35,974	15.4 (10.9–21.4)	16,032	41.4 (31.9–51.6)
No	262,759	14.9 (12.7–17.3)	129,156	10.9 (8.2–14.4)	88,221	10.4 (7.7–13.8)	45,382	34.8 (27.1–43.5)

Source: National Health Survey of Panama (ENSPA) 2019; CI: confidence interval; “stunting” and “wasting” were defined according to the cut-off points of the World Health Organization Growth Standards; Undernutrition: wasting only, stunting only, or wasting and stunting.

Table 3 presents the weighted prevalence of overnutrition among children under age five by baseline characteristics at the national level and by living area. According to the living area, the prevalence of overnutrition was higher among children in urban areas (11.9%; 95% CI: 8.5–16.3) than among those in both rural areas (8.4%; 95% CI: 6.5–10.7) and indigenous areas (8.7%; 95% CI: 5.2–14.3). Children under 12 months old presented the highest prevalence of overnutrition (21.1%; 95% CI: 13.9–30.6). At the national level, there were no differences by sex in the prevalence of overnutrition. However, in indigenous areas, overnutrition was more prevalent among male (12.9%; 95% CI: 6.8–23.1) than female (4.6%; 95% CI: 2.3–9.1) children. Overall, overnutrition was less prevalent among children with unimproved drinking water sources and those with episodes of diarrhoea in the last two weeks.

**Table 3:** Weighted prevalence of overnutrition among children under five by baseline characteristics at the national level and by living area, Panama, 2019.

SELECTED BASELINE CHARACTERISTICS	LIVING AREA							
	NATIONAL		URBAN AREA		RURAL AREA		INDIGENOUS AREA	
	N	% (95% CI)	N	% (95% CI)	N	% (95% CI)	N	% (95% CI)
Total	370,408	10.2 (8.2–12.6)	184,798	11.9 (8.5–16.3)	124,196	8.4 (6.5–10.7)	61,414	8.7 (5.2–14.3)
**Sociodemographic characteristics**								
**Age (months)**								
0–11	50,317	21.1 (13.9–30.6)	24,303	27.5 (15.8–43.5)	17,228	8.1 (4.2–14.8)	8,786	28.9 (13.1–52.2)
12–23	75,137	10.1 (5.1–18.8)	36,031	11.7 (3.7–31.6)	25,582	11.6 (7.1–18.2)	13,524	3.0 (0.8–10.5)
24–35	85,444	5.7 (3.9–8.2)	38,879	5.9 (3.3–10.4)	28,759	6.1 (3.5–10.3)	17,805	4.4 (1.6–11.4)
36–47	80,472	12.3 (8.8–16.9)	46,949	12.4 (7.6–19.6)	24,490	12.0 (7.4–18.8)	9,033	12.4 (4.8–28.3)
48–59	79,038	6.1 (4.0–9.1)	38,635	7.6 (4.3–13.0)	28,136	4.8 (2.5–9.1)	12,266	4.2 (1.2–14.2)
**Sex**								
Male	201,250	10.0 (7.5–13.0)	108,778	10.3 (6.8–15.3)	62,085	8.0 (5.6–11.3)	30,386	12.9 (6.8–23.1)
Female	169,158	10.4 (7.4–14.5)	76,020	14.2 (8.5–22.6)	62,110	8.7 (6.2–12.3)	31,027	4.6 (2.3–9.1)
**Overcrowding**								
Yes	53,454	10.9 (6.5–17.6)	15,436	13.2 (5.7–27.8)	12,737	9.0 (3.9–19.6)	25,281	10.4 (4.4–22.9)
No	316,953	10.0 (7.9–12.7)	169,362	11.8 (8.3–16.5)	111,458	8.3 (6.4–10.7	36,133	7.5 (4.2–13.0)
**Household monthly income quartile (USD; $)**								
First quartile (≤124)	101,824	10.1 (7.1–14.1)	18,679	14.3 (6.4–29.2)	37,233	9.5 (6.5–13.8)	45,911	8.8 (4.7–15.9)
Second quartile (125–399)	94,054	12.6 (7.6–20.3)	45,205	18.2 (9.1–33.2)	37,469	7.3 (4.7–11.1)	11,380	8.0 (2.5–22.7)
Third quartile (400–699)	83,150	9.6 (6.7–13.6)	54,117	9.6 (6.1–14.9)	27,478	9.6 (5.1–17.3)	1,555	10.9 (1.6–48.5)
Fourth quartile (≥700)	69,765	9.1 (6.0–13.6)	55,405	9.6 (5.9–15.3)	14,064	7.2 (3.8–13.3)	296	5.3 (0.7–32.1)
**Household dietary diversity**								
Low (0–3)	28,576	11.1 (5.9–19.7)	7,653	16.1 (4.2–45.9)	6,182	13.5 (5.6–29.1)	14,740	7.5 (3.1–16.8)
Medium (4–6)	63,164	7.7 (5.2–11.4)	21,696	8.6 (4.4–16.2)	19,627	6.2 (3.3–11.4)	21,840	8.1 (3.8–16.4)
High (7–12)	278,668	10.6 (8.2–13.7)	155,449	12.1 (8.4–17.2)	98,386	8.5 (6.4–11.2)	24,833	10.0 (4.0–22.9)
**WASH characteristics**								
**Drinking water source status**								
Unimproved	47,651	6.0 (3.8–9.4)	8,206	8.2 (3.1–20.1)	16,649	5.1 (2.4–10.5)	22,797	5.8 (2.8–11.6)
Improved	322,756	10.8 (8.6–13.5)	176,592	12.0 (8.6–16.6)	107,548	8.9 (6.8–11.5)	38,617	10.4 (5.5–19.0)
**Sanitation system**								
Unimproved	127,008	8.9 (6.6–11.9)	22,327	11.4 (6.5–19.3)	47,197	8.4 (5.8–12.0)	57,484	8.4 (4.8–14.3)
Improved	243,303	10.8 (8.2–14.2)	162,375	11.9 (8.3–16.9)	76,999	8.3 (6.0–11.5)	3,929	13.6 (4.0–37.2)
**Adequate Hygiene**								
Yes	133,514	10.9 (7.3–16.0)	71,314	11.0 (5.7–20.3)	48,206	9.5 (6.5–13.6)	13,994	14.9 (5.1–36.4)
No	153,547	7.9 (5.8–10.6)	75,800	10.0 (6.4–15.1)	46,406	5.8 (3.9–8.7)	31,340	6.0 (3.1–11.4)
**Access to health care**								
**Adequate growth checkups**								
Yes	285,738	9.8 (7.6–12.5)	143,061	11.7 (8.0–16.7)	97,052	8.6 (6.5–11.5)	45,624	6.4 (3.7–10.9)
No	84,670	11.4 (7.3–17.5)	41,737	12.6 (6.3–23.6)	27,143	7.3 (4.7–11.3)	15,790	15.5 (6.2–33.6)
**Comorbidities**								
**Diarrhoea**								
Yes	37,347	5.8 (3.3–9.9)	14,129	5.5 (1.9–15.0)	11,123	2.7 (0.9–8.0)	12,095	8.8 (4.1–18.1)
No	325,154	10.5 (8.4–13.2)	166,754	12.4 (8.8–17.2)	110,387	8.5 (6.5–10.9)	48,013	8.9 (4.8–16.0)
**Acute respiratory infections**								
Yes	107,648	10.8 (6.3–17.8)	55,642	14.9 (7.2–28.1)	35,974	6.3 (3.9–10.3)	16,032	6.4 (2.8–13.8)
No	262,759	9.9 (8.1–12.1)	129,156	10.6 (7.9–14.1)	88,221	9.2 (6.9–12.1)	45,382	9.5 (5.1–17.0)

Source: National Health Survey of Panama (ENSPA) 2019; CI: confidence interval; “overweight” and “obesity” were defined according to the cut-off points of the World Health Organization Growth Standards; “overnutrition”: overweight only or obesity only.

Table 4 presents the weighted prevalence of DBM among children under age five by baseline characteristics at the national level and by living area. The prevalence of DBM in indigenous areas (2.7%; 95% CI: 1.4–5.1) was almost double that at the national level (1.4%; 95% CI: 1.0–2.1), followed by rural areas (1.6%; 95% CI: 0.8–2.9). At the national level, children under 12 months old (3.2%; 95% CI: 1.7–5.9) and those with unimproved sanitation systems (2.6%; 95% CI: 1.6–4.0) presented the highest prevalence of DBM. In the urban area, children without adequate growth checkups (1.9%; 95% CI: 0.8–4.8) presented a higher prevalence of DBM than those who had adequate growth checkups (0.6%; 95% CI: 0.3–1.6). On the other hand, in rural areas, children living in overcrowded households presented the highest prevalence of DBM (6.7%; 95% CI: 2.2–18.7).

**Table 4:** Weighted prevalence of the double burden of malnutrition among children under age five by baseline characteristics at the national level and by living area, Panama, 2019.

SELECTED BASELINE CHARACTERISTICS	LIVING AREA							
	NATIONAL		URBAN AREA		RURAL AREA		INDIGENOUS AREA	
	N	% (95% CI)	N	% (95% CI)	N	% (95% CI)	N	% (95% CI)
Total	370,408	1.4 (1.0–2.1)	184,798	0.9 (0.5–1.8)	124,196	1.6 (0.8–2.9)	61,414	2.7 (1.4–5.1)
**Sociodemographic characteristics**								
**Age (months)**								
0–11	50,317	3.2 (1.7–5.9)	24,303	3.3 (1.3–8.1)	17,228	4.4 (1.8–10.2)	8,786	0.4 (0.1–1.8)
12–23	75,137	2.1 (1.0–4.1)	36,031	2.1 (0.7–6.0)	25,582	1.5 (0.5–3.9)	13,524	3.1 (0.7–12.0)
24–35	85,444	1.2 (0.5–3.1)	38,879	0.0	28,759	1.8 (0.4–7.8)	17,805	2.9 (0.9–8.8)
36–47	80,472	0.7 (0.2–1.9)	46,949	0.0	24,490	0.1 (0.0–0.7)	9,033	5.6 (1.9–15.8)
48–59	79,038	0.8 (0.2–2.4)	38,635	0.4 (0.1–1.7)	28,136	1.1 (0.1–7.2)	12,266	1.4 (0.2–9.5)
**Sex**								
Male	201,250	1.8 (1.2–2.7)	108,778	1.3 (0.6–2.6)	62,085	1.7 (0.8–3.3)	30,386	4.0 (1.9–8.1)
Female	169,158	1.0 (0.5–2.1)	76,020	0.4 (0.1–1.8)	62,110	1.5 (0.5–4.3)	31,027	1.4 (0.3–5.3)
**Overcrowding**								
Yes	53,454	2.4 (1.0–5.4)	15,436	1.3 (0.2–6.5)	12,737	6.7 (2.2–18.7)	25,281	0.9 (0.2–4.0)
No	316,953	1.3 (0.8–1.9)	169,362	0.9 (0.4–1.8)	111,458	1.0 (0.5–2.0)	36,133	3.9 (1.9–8.0)
**Household monthly income quartile (USD; $)**								
First quartile (≤124)	101,824	2.3 (1.4–4.0)	18,679	0.6 (0.1–3.8)	37,233	2.6 (1.1–6.0)	45,911	2.8 (1.4–5.8)
Second quartile (125–399)	94,054	1.2 (0.6–2.5)	45,205	1.1 (0.3–3.4)	37,469	0.8 (0.2–3.0)	11,380	3.1 (2.1–11.5)
Third quartile (400–699)	83,150	0.7 (0.2–2.2)	54,117	1.1 (0.3–3.3)	27,478	0.0	1,555	0.0
Fourth quartile (≥700)	69,765	1.4 (0.5–3.9)	55,405	0.8 (0.2–3.4)	14,064	3.7 (0.8–15.3)	296	0.0
**Household dietary diversity**								
Low (0–3)	28,576	2.9 (1.2–7.0)	7,653	2.1 (0.3–14.7)	6,182	2.9 (0.4–17.9)	14,740	3.4 (1.1–10.1)
Medium (4–6)	63,164	1.9 (0.9–3.9)	21,696	0.5 (0.1–3.3)	19,627	1.6 (0.5–5.1)	21,840	3.6 (1.3–9.3)
High (7–12)	278,668	1.2 (0.7–1.9)	155,449	0.9 (0.4–1.9)	98,386	1.5 (0.7–3.2)	24,833	1.5 (0.4–5.3)
**WASH characteristics**								
**Drinking water source status**								
Unimproved	47,651	1.2 (0.4–3.0)	8,206	0.0	16,649	1.2 (0.3–4.2)	22,797	1.5 (0.4–5.8)
Improved	322,756	1.5 (1.0–2.2)	176,592	1.0 (0.5–1.8)	107,548	1.6 (0.8–3.2)	38,617	3.4 (1.6–6.9)
**Sanitation system**								
Unimproved	127,008	2.6 (1.6–4.0)	22,327	2.0 (0.5–7.0)	47,197	2.5 (1.2–5.2)	57,484	2.8 (1.5–5.4)
Improved	243,303	0.8 (0.4–1.6)	162,375	0.8 (0.4–1.7)	76,999	1.0 (0.3–3.1)	3,929	0.3 (0.0–2.6)
**Adequate Hygiene**								
Yes	133,514	0.7 (0.3–1.5)	71,314	0.6 (0.2–2.0)	48,206	0.6 (0.2–2.4)	13,994	1.1 (0.2–7.7)
No	153,547	1.5 (0.8–2.8)	75,800	0.3 (0.1–1.8)	46,406	1.7 (0.5–5.6)	31,340	4.2 (2.0–8.6)
**Access to health care**								
**Adequate growth checkups**								
Yes	285,738	1.2 (0.8–1.9)	143,061	0.6 (0.3–1.6)	97,052	1.3 (0.5–2.9)	45,624	3.0 (1.5–5.9)
No	84,670	2.2 (1.2–3.9)	41,737	1.9 (0.8–4.8)	27,143	2.7 (1.1–6.6)	15,790	1.8 (0.3–9.4)
**Comorbidities**								
**Diarrhoea**								
Yes	37,347	1.7 (0.6–4.5)	14,129	0.0	11,123	1.0 (0.1–6.5)	12,095	4.4 (1.4–12.8)
No	325,154	1.4 (1.0–2.1)	166,754	1.0 (0.5–2.0)	110,387	1.7 (0.9–3.2)	48,013	2.3 (1.1–5.1)
**Acute respiratory infections**								
Yes	107,648	1.0 (0.4–2.3)	55,642	0.3 (0.1–1.3)	35,974	1.3 (0.3–5.3)	16,032	2.6 (0.6–10.0)
No	262,759	1.6 (1.1–2.4)	129,156	1.2 (0.6–2.4)	88,221	1.7 (0.8–3.4)	45,382	2.7 (1.3–5.5)

Source: National Health Survey of Panama (ENSPA) 2019; CI: confidence interval; “stunting,” “overweight,” and “obesity” were defined according to the cut-off points of the World Health Organization Growth Standards; “double burden”: stunting and overweight/obesity.

## Discussion

This study estimated the prevalence of undernutrition, overnutrition, and DBM among children under age five at the national level and by living area in Panama.

The total prevalence of malnutrition among children under age five in Panama has marginally changed since 2008 (from 27.1% in 2008 to 26.9% in 2019) [8]. However, there have been changes in the prevalence of different forms of malnutrition.

Undernutrition is the most prevalent malnutrition burden worldwide, in Latin America, as well as in Panama [2, 19]. The prevalence of undernutrition in Panama has decreased from 17.4% to 15.3% (2.1 percentage points) since 2008. The prevalence was similar to those of other countries in the region, such as Mexico (13.9%) in 2021, El Salvador (14.8%) in 2014, Nicaragua (17.4%) in 2012, and Colombia (13.3%) in 2016 [19].

Despite the slight decrease in the national undernutrition prevalence, undernutrition in indigenous areas of Panama remains an important public health problem. Although most studies consider only urban and rural areas and not indigenous areas as living areas, indigenous populations are also studied as ethnic groups. Similar to our findings, in Guatemala, Ecuador, Mexico, and Peru, indigenous children presented the highest prevalence of stunting and wasting, according to nationally representative surveys [20–23]. Additionally, in Chiapas, Mexico, children of indigenous mothers presented a higher probability of stunting [24]. A multicentric study carried out among children under age five from six countries worldwide to develop the WHO growth standards indicated that under optimal environmental conditions, children’s growth expectancy is the same, regardless of ethnicity [25]. Hence, socioeconomic disparities and cultural factors may be responsible for undernutrition problems in these groups.

Similar to our findings, in the study carried out in Chiapas, Mexico, children <12 months presented a lower likelihood of stunting than those between 12 and 59 months [24]. Additionally, similar to what was observed in indigenous areas of Panama, in a study carried out among indigenous children of a rural area of Ecuador (Chimborazo Province), children aged under 12 months presented better height-for-age Z scores [26]. As age increases, nutritional needs also increase [27], leading to a higher risk of not meeting the nutritional requirements of the diet.

Globally, income has been described as one of the main determinants of undernutrition [28, 29]. Although Panama’s economic growth has recently reached a high-income status based on gross domestic product in 2019, income disparities in the same year were among the highest in the CELAC region, according to the latest Gini Index [30]. Because most of the children in indigenous areas of Panama live in a household in the lowest monthly household income quartile, this population requires special attention regarding the undernutrition burden.

In the present study, undernutrition was more prevalent among children in overcrowded households regardless of living area. Similar to our results, in the study carried out in Chimborazo, Ecuador, families without overcrowding presented better height-for-age Z scores [26]. Several studies in Pakistan have also shown overcrowding or large family size to be a determinant factor of child malnutrition [31]. Food distribution among family members may also be unequal, favouring family heads, which in turn leads to food amounts not fulfilling the children’s energy requirements [32], especially in overcrowded households where food security is lacking [33]. This might be yet another factor that was common among children living in indigenous areas, according to our study.

A higher HDD indicates better food access and thus better nutrition among family members, [34] which could directly influence children’s nutrition and dietary diversity [35], especially in preschool children, whose caregivers have a high impact on their nutritional practices. In our study, children with a high HDD presented the lowest prevalence of undernutrition. Such is also the case in East Java Province in Indonesia, where for a 1-point increase in HDD score, the probability of stunting decreased by more than 10% [36]. In a cross-sectional study that included 35 low- and middle-income countries, HDD was also found to be associated with undernutrition, placing fifth and third among 20 factors analysed associated with stunting and wasting, respectively [37].

In the same study, but carried out in Indonesia, WASH factors such as unimproved sanitation and unsafe stool disposal were found to be associated with stunting, whereas unimproved sanitation and unsafe water were associated with wasting [37]. It has been pointed out that improving rates of open defecation and sanitation infrastructure accounted for substantial improvement in child growth globally [28]. Furthermore, WASH factors are important to maintaining health and preventing infectious diseases and diarrhoea morbidity and mortality [38]. Diarrhoea leads to gut inflammation and thus reduced nutrient absorption, which, consequently, may affect children’s growth and nutritional status [39, 40]. In line with this, our study showed the highest prevalence of undernutrition among children with unimproved or inadequate WASH factors and with episodes of diarrhoea in the previous 2 weeks.

The prevalence of overnutrition, on the other hand, increased from 7.2% in 2008– to 10.2% (three percentage points). It was higher than in other countries of the Central America Region, according to their latest estimate. The prevalence of overnutrition in Panama was also higher than that in Mexico (6.8%) in 2021, in Ecuador (7.4%) in 2019, and in Colombia (4.8%) in 2016 [19].

Overall, the prevalence of overnutrition was the highest in the urban area and slightly higher in indigenous areas than in rural areas. Similarly, in a nationally representative study in Mexico, indigenous children presented a higher prevalence of overweight and obesity than nonindigenous children, and children living in urban areas had a higher prevalence than those in rural areas [23].

The prevalence of overnutrition tends to increase with age [41]. However, our study showed the highest prevalence of overnutrition occurring among children in the youngest age group (children aged under a year) at the national level and in urban and indigenous areas. Similar to the findings of our study, in East African countries, children under 24 months old presented higher odds of overnutrition than did older children [42].

A previous study carried out in low- and middle-income countries showed a higher prevalence of overweight in the highest income quintiles than in the lowest quintiles [1]. Similarly, in a study conducted in the Latin America and Caribbean (LAC) region, a positive association was found between wealth and overweight [29]. In contrast, in high-income countries, an inverse association between income and overweight has been described [43]. However, in our study, no significant differences in overnutrition prevalence were observed between the household income quartiles. The fact that Panama has only recently been classified as a high-income country may explain this finding [30].

The DBM has gained interest in recent years because it has been associated with recent and a marked shifts in the industrialization of developing countries [9]. When comparing 13 LAC region countries with DBM data from 2018 to 2020, the prevalence of DBM in Panama (1.4%) was higher than in nine other countries and lower only than Uruguay (1.6%), Turks and Caicos (3.7%), and Ecuador (4.4%) [19].

Although the prevalence of DBM was lower than the prevalence of undernutrition and overnutrition, it was the highest in indigenous areas, followed by rural areas. In agreement with our findings, in a population-based study in Guatemala, DBM at the individual level was higher in indigenous (2.8%) than in nonindigenous children (1.2%) [44]. Additionally, in a study carried out in two metropolitan regions in Bolivia, DBM was higher in peri-urban and rural areas than in urban areas [45]. In our study, DBM was found to be more prevalent in younger children, similar to what was found in a review by Steyn and Nel, where DBM was more prevalent among children under 24 months than among those aged 24–59 months [46].

In terms of income, in the LAC region, DBM has been associated with the lowest wealth quintile [29]. However, variable results have been observed in different countries and regions [29, 46]. The authors argue that the discrepancies in these associations may be due to different stages of the nutrition transition or to the low prevalence of this condition [29]. In our study, the prevalence of DBM was not associated with household income, a finding similar to that found in a Bolivian study [45].

Economic growth and urbanization are important drivers of the nutrition transition, which is characterized by a change in dietary patterns that includes an increased intake of ultra-processed, energy-dense, and micronutrient-deficient food [47]. In the LAC region, ultra-processed-food sales per capita have been constantly increasing. Furthermore, ultra-processed-beverage sales per capita in the region were among the highest in 2019, following the Australasian, North American, and Western European regions [47]. These changes in dietary patterns have resulted in increased overweight and obesity rates, as well as rates of non-communicable diseases worldwide among all ages [48]. This, combined with the still-prevalent undernutrition problem, especially in children under age five, causes an increased prevalence of the DBM. Furthermore, undernourished children exposed to these drastic changes in nutrition may present a DBM at the individual level, leading to the development of non-communicable diseases in adulthood [7]. Our study showed a higher prevalence of DBM with a decrease in HDD.

As part of the initiatives to prevent and treat undernutrition, Panama has a Maternal–Child Complementary Feeding Program created for children from age six to age 59 months and for pregnant and lactating women that includes the distribution of a pre-cooked and enriched nutritional complement to individuals at nutritional-deficit risk. However, it is important to continue monitoring these types of programs because the unequal household distribution of food brought in this program could lead to DBM both individually and at the household level. On the other hand, programs aiming to prevent overnutrition are mostly focused on children aged 5 years or older and on adults, and to our knowledge, the DBM has not yet been taken into consideration in any nutritional program. It is thus essential to assess both ends of the malnutrition burden when developing national policies targeting this vulnerable population, for whom both deficit and excess may carry lifelong consequences. Given the important socioeconomic disadvantages observed in the indigenous population and the higher prevalence of both undernutrition and DBM, a special focus is needed in this population.

## Limitations

This study has several limitations. Intra- and interevaluator variability in the measurement of anthropometric data may be a source of bias. However, evaluators were trained on the standardized measuring techniques, and extreme values on anthropometric variables were excluded. There is also a potential bias caused by the number of missing values. However, the age, sex, and area of excluded children are described in the supplementary materials. Unlike this study, in which the weight-for-height/length indicator was used, some studies used the BMI-for-age indicator to determine overweight and obesity nutritional status [21, 22, 45, 46], affecting the precision of the comparison. Nevertheless, the WHO and the United Nations International Children’s Emergency Fund (UNICEF) recommend using the weight-for-height/length indicator when assessing overweight and obesity in children under age five [49]. Even though there were queries regarding breastfeeding and complementary feeding practices in ENSPA, they were not included in the current study because they were applicable only to subgroups of certain ages in our study sample. Finally, the cross-sectional nature of the data does not allow us to explore temporal trends or to evaluate causality.

## Strengths

The results presented in this study are nationally representative. The use of a nationally representative sample allows comparisons with other countries that used similar methodologies. This is the first nationally and subnationally representative study evaluating the DBM together with possible factors, thus laying the foundation on which to develop, strengthen, and update national nutritional policies for children under age five.

## Conclusions

Our results indicate that undernutrition, particularly stunting, in children under age five is still the most prevalent type of malnutrition in our country, especially in indigenous areas. The prevalence of overnutrition was higher than that in all other countries in Central America. Even though the prevalence of DBM was low, it should be monitored, considering the nutrition transition to which economic growth and industrialization has led us and the great inequalities present in our population. Further research is necessary to better understand the aetiology of the DBM.

## Data Sharing Statement

General results from the ENSPA study are publicly available in the Spanish language and can be downloaded from the following web link: https://www.gorgas.gob.pa/wp-content/uploads/external/SIGENSPA/Inicio.htm.

## Additional File

The additional file for this article can be found as follows:

Supplementary File 1. Table 1. Baseline characteristics of excluded children under five, Panama, 2019.

Supplementary File 2. Table 2. Weighted prevalence of stunting and/or wasting among children under five by baseline characteristics, Panama, 2019.
