# Supplementary material for: Malnutrition among Children under Age Five in Panama: Results of the ENSPA 2019
Source: Ann Glob Health. 2024 Aug 8;90(1):51. doi: 10.5334/aogh.4409 (PMC11312709; doi:10.5334/aogh.4409)
Supplement: Supplementary File 1. — Table 1. Baseline characteristics of excluded children under five, Panama, 2019. [file agh-90-1-4409-s1.pdf]

Supplementary Table 1. Baseline characteristics of excluded children under five, Panama, 2019.

Selected baseline characteristics	National	
**Sociodemographic characteristics**	**N**	**Weighted prevalence % (95% CI)**
**Age (months)**		
0–11	14,627	14.2 (10.1–19.7)
12–23	22,285	21.7 (16.7–27.6)
24–35	18,679	18.2 (14.4–22.6)
36–47	25,668	25.0 (20.1–30.6)
48–59	21,571	21.0 (15.9–27.1)
**Female sex**	49,272	47.9 (41.7–54.2)
**Living area**		
Urban	67,542	65.7 (60.1–70.9)
Rural	30,647	29.8 (25.0–35.1)
Indigenous	4,641	4.5 (2.9–7.0)

Source: National Health Survey of Panama (ENSPA) 2019; CI: confidence interval.
