# Supplementary material for: Malnutrition among Children under Age Five in Panama: Results of the ENSPA 2019
Source: Ann Glob Health. 2024 Aug 8;90(1):51. doi: 10.5334/aogh.4409 (PMC11312709; doi:10.5334/aogh.4409)
Supplement: Supplementary File 2. — Table 2. Weighted prevalence of stunting and/or wasting among children under five by baseline characteristics, Panama, 2019. [file agh-90-1-4409-s2.pdf]

Supplementary Table 2. Weighted prevalence of stunting and/or wasting among children under five by baseline characteristics, Panama, 2019.

	Undernutrition		
**Selected baseline characteristics**	**Wasting only**	**Stunting only**	**Stunting and wasting**
	**% (95% CI)**	**% (95% CI)**	**% (95% CI)**
Total (N=370,408)	0.7 (0.4–1.2)	14.4 (12.6–16.3)	0.2 (0.1–0.7)
**Sociodemographic characteristics**			
**Age (months)**			
0–11 (N=50,317)	2.2 (0.8–5.6)	7.2 (4.5–11.1)	0.2 (0.0–1.6)
12–23 (N=75,137)	1.1 (0.4–3.0)	16.8 (13.0–21.5)	0.7 (0.1–3.8)
24–35 (N=85,444)	0.2 (0.0–1.0)	17.2 (13.3–21.9)	0.2 (0.0–0.8)
36–47 (N=80,472)	0.2 (0.0–0.8)	14.0 (10.4–18.6)	0.0 (0.0–0.2)
48–59 (N=79,038)	0.3 (0.1–1.1)	14.0 (10.3–18.6)	0.0 (0.0–0.0)
**Sex**			
Male (N=201,250)	0.6 (0.3–1.3)	15.5 (13.0–18.5)	0.4 (0.1–1.3)
Female (N=169,158)	0.8 (0.3–1.8)	13.0 (10.8–15.6)	0.0 (0.0–0.1)
**Living area**			
Urban (N=184,798)	0.6 (0.2–1.3)	9.7 (7.5–12.5)	0.3 (0.0–1.8)
Rural (N=124,196)	1.1 (0.5–2.4)	10.6 (8.4–13.3)	0.2 (0.1–0.7)
Indigenous (N=61,414)	0.2 (0.1–0.9)	36.1 (29.8–43.0)	0.2 (0.0–0.8)
**Overcrowding**			
Yes (N=53,454)	0.2 (0.0–1.1)	29.3 (24.3–35.0)	0.2 (0.0–1.0)
No (N=316,953)	0.7 (0.4–1.4)	11.8 (10.0–13.9)	0.2 (0.1–0.9)
**Household monthly income quartile (USD; $)**			
First quartile (≤124) (N=101,824)	0.5 (0.2–1.2)	24.6 (20.6–29.1)	0.0 (0.0–0.1)
Second quartile (125–399) (N=94,054)	1.2 (0.5–3.1)	12.8 (9.9–16.3)	0.3 (0.1–0.8)
Third quartile (400–699) (N=83,150)	0.5 (0.1–2.6)	10.9 (7.5–15.7)	0.0 (0.0–0.0)
Fourth quartile (≥700) (N=69,765)	0.3 (0.1–1.7)	5.9 (3.6–9.5)	0.7 (0.1–4.6)
**Household dietary diversity**			
Low (0–3) (N=28,576)	0.6 (0.1–3.0)	24.5 (17.2–33.6)	0.0 (0.0–0.0)
Medium (4–6) (N=63,164)	0.1 (0.0–0.3)	23.5 (18.5–29.3)	0.2 (0.0–0.9)
High (7–12) (N=278,668)	0.8 (0.4–1.5)	11.3 (9.4–13.4)	0.3 (0.1–1.0)

**WASH characteristics**			
**Drinking water source status**			
Unimproved (N=47,651)	1.9 (0.6–5.8)	23.3 (18.1–29.5)	0.1 (0.0–0.3)
Improved (N=322,756)	0.5 (0.3–0.9)	13.1 (11.2–15.1)	0.2 (0.1–0.8)
**Sanitation system**			
Unimproved (N=127,008)	0.9 (0.4–2.3)	24.2 (20.8–28.1)	0.2 (0.1–0.6)
Improved (N=243,303)	0.5 (0.3–1.1)	9.2 (7.4–11.5)	0.2 (0.0–1.2)
**Adequate hygiene**			
Yes (N=133,514)	0.5 (0.1–1.6)	11.8 (9.3–15.0)	0.0 (0.0–0.0)
No (N=153,547)	0.4 (0.2–0.8)	15.9 (13.1–19.2)	0.4 (0.1–1.9)
**Access to health care**			
**Adequate growth checkups**			
Yes (N=285,738)	0.6 (0.3–1.1)	14.4 (12.4–16.7)	0.3 (0.1–0.9)
No (N=84,670)	0.9 (0.3–3.2)	14.2 (11.0–18.3)	0.1 (0.0–0.9)
**Comorbidities**			
**Diarrhoea**			
Yes (N=37,347)	0.8 (0.2–3.4)	26.1 (19.0–34.9)	0.3 (0.0–2.1)
No (N=325,154)	0.6 (0.3–1.2)	13.0 (11.3–14.9)	0.2 (0.1–0.8)
**Acute respiratory infections**			
Yes (N=107,648)	0.6 (0.2–1.4)	15.1 (12.0–18.8)	0.5 (0.1–2.6)
No (N=262,759)	0.7 (0.3–1.4)	14.1 (12.0–16.4)	0.1 (0.0–0.3)

Source: National Health Survey of Panama (ENSPA) 2019; CI: confidence interval; Stunting and wasting were defined according to the cut-off points of the World Health Organization Growth Standards; Undernutrition: wasting only, stunting only or wasting and stunting.
